# BCG Vaccine Protection against TB Infection among Children Older than 5 Years in Close Contact with an Infectious Adult TB Case

**DOI:** 10.3390/jcm9103224

**Published:** 2020-10-08

**Authors:** Angeliki Syggelou, Nikolaos Spyridis, Kyriaki Benetatou, Eleni Kourkouni, Georgia Kourlaba, Maria Tsagaraki, Despoina Maritsi, Irini Eleftheriou, Maria Tsolia

**Affiliations:** 1 Second Department of Paediatrics, National and Kapodistrian University of Athens, School of Medicine, ‘P. and A. Kyriakou’ Children’s Hospital, 30601 Athens, Greece; asyggelou@yahoo.gr (A.S.); nspyridis@hotmail.co.uk (N.S.); kiriakiben@yahoo.gr (K.B.); ts.maria@yahoo.gr (M.T.); dmaritsi@gmail.com (D.M.); eleftheriou.eir@gmail.com (I.E.); 2 Center for Clinical Epidemiology and Outcomes Research (CLEO), Nonprofit Civil Partnership, 30601 Athens, Greece; e.kourkouni@cleoresearch.org (E.K.); g.kourlaba@cleoresearch.org (G.K.)

**Keywords:** tuberculosis, BCG, contacts, children, latent TB infection

## Abstract

The Bacille Calmette–Guérin (BCG) vaccine has been shown to provide considerable protection against miliary or meningeal tuberculosis (TB), but whether it prevents other forms of disease remains controversial. Recent evidence has shown that the BCG vaccine also provides protection against latent TB infection (LTBI). The aim of the current study was to examine whether BCG has a protective role against LTBI among children in close contact with an adult index case in a low TB endemicity setting with the use of the QuantiFERON-TB Gold In-Tube test (QFT-GIT). A cross-sectional study was conducted over a 10-year period among children referred to our outpatient TB clinic with a history of close contact with an adult with pulmonary TB. All subjects had a QFT-GIT performed. In total, 207 children > 5 to 16 years of age with known recent exposure were enrolled. BCG-vaccinated subjects had a 59% lower risk of presenting with LTBI after close contact with an adult index case compared with unvaccinated subjects (OR = 0.41, 95% CI: 0.23–0.73, *p* = 0.002). After adjustment for possible confounders, the protective effect of prior BCG immunization was estimated at 68% (OR = 0.32, 95% CI: 0.15–0.66, *p* = 0.002). Other risk factors for LTBI included a history of migration (OR = 2.27, 95% CI: 1.13–4.53, *p* = 0.021) and transmission of infection to other exposed child contacts (OR = 4.62, 95% CI: 2.27–9.39, *p* = 0.001). We were able to determine a strong protective role of BCG vaccination among children older than 5 years, immunized at school entry, who had close contact with an adult infectious TB case.

## 1. Introduction

The Bacille Calmette–Guérin (BCG) vaccine is currently the only licensed vaccine against tuberculosis (TB) and is included in the childhood immunization program in many countries. This vaccine has been in use since 1921 and is the most widely used vaccine, with an estimated 100 million doses administered to neonates annually worldwide [1]. The vaccine’s efficacy against pulmonary disease has been found to range from 0% to 80% and varies in different geographical areas. Previous nontuberculous mycobacterial exposure influences vaccine efficacy and has been shown to account, at least in part, for the observed differences [2]. BCG has been shown to provide considerable protection against miliary or meningeal TB in randomized controlled trials and in case-control studies [3]. When administered to neonates and infants, BCG reduces the risk of TB by more than 50% and protects against severe and disseminated forms of the disease [2,4]. Although BCG provides protection against progression from TB infection to active disease, it was, until recently, under question whether this vaccine protects exposed individuals from acquiring TB infection [5,6,7]. With the recent development of the interferon-γ release assays (IGRAs), it has become possible to diagnose TB infection with higher accuracy compared to the tuberculin skin test (TST) since these assays have higher specificity and can discriminate between TB infection and previous BCG immunization or infection with most environmental mycobacteria [8]. With the use of IGRAs, a number of studies have shown a protective effect of BCG immunization against latent TB infection (LTBI) [9,10,11], whereas others did not [12]. In a previous multicenter study conducted with the participation of five different European sites, including our center, a large number of children evaluated for LTBI were examined both with TST and at least one IGRA [13]. It was shown that BCG protects exposed children from acquiring *Mycobacterium tuberculosis* infection. In a recent meta-analysis of studies that examined BCG-vaccinated and unvaccinated children who were exposed to TB with the use of IGRAs, it was shown that BCG protects against *M. tuberculosis* infection, as well as from progression to active disease [14]. New cases of childhood TB usually arise after contact with an infectious adult index case, and data highlight that, in the absence of intervention, up to 40% of children less than 2 years of age who have been infected will progress to active disease [13].

The aim of the current study was to examine whether BCG vaccination has a protective role against latent TB infection (LTBI) among children in close contact with an adult index case in a low TB endemicity setting with the use of the QuantiFERON-TB Gold In-Tube test (QFT-GIT). The secondary objective of the study was to examine other possible risk factors of LTBI among children exposed to an adult with infectious TB.

Greece is a low-incidence country with 4–5 cases of TB reported annually per 100,000 people, although the actual number may be much higher [15,16]. It has been estimated that about 40% of the reported cases occur among people of foreign origin [15]. Our TB clinic is a reference center for pediatric TB cases in central and parts of southern Greece, and we, therefore, had the opportunity to examine a considerable number of children exposed to an infectious adult case.

## 2. Experimental Section

### 2.1. Subjects

**Figure 1:**
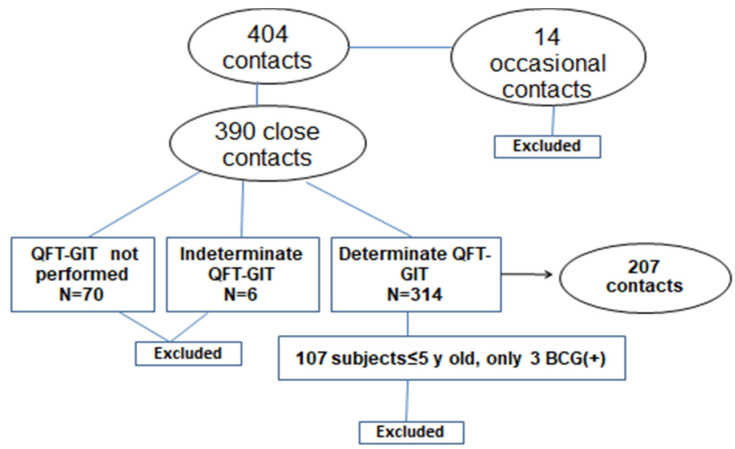
Flow diagram with patients enrolled in the study.

Subjects who had a positive contact history with an adult treated for pulmonary TB were recruited from our outpatient TB clinic, where they were evaluated for LTBI or active TB over a 10-year period between 1 January 2007 and 31 December 2016. This cohort study included both retrospective and prospective phases. During the first 3 years of the study, data were collected retrospectively; this was followed by a 7-year period of prospective evaluation of the identified cohorts (2010–2016). Children who met the following criteria were included: (1) age ≤ 16 years, (2) known recent close contact with an adult with pulmonary TB, (3) known BCG status with either the presence of a scar and/or the availability of an immunization record, (4) documented result of QFT-GIT and (5) normal physical examination and chest X-ray. During the study period, all patients evaluated for active TB or TB infection had a QFT-GIT performed. Only close contacts, defined as those living in the same household or in frequent contact for at least 6–9 h per week with an adult pulmonary TB source case, were included. Patients were considered to have TB infection if the QFT-GIT result was positive in the absence of symptoms or physical findings compatible with TB and normal chest X-ray. Participants with indeterminate QFT-GIT results were excluded from the study. Contacts found to have active TB during the evaluation were not included in the study (Figure 1). The TST was considered positive if the induration was ≥ 5 mm. Contacts with a negative early (<10 weeks after last contact with the index case) QFT-GIT were re-tested at ≥ 10 weeks after the last exposure to the index case while infectious. In these cases, only this second testing was recorded. The laboratory researcher performing the QFT-GIT assay was blinded with regard to individual patients’ clinical data.

At the baseline visit, a standardized questionnaire was filled out for each participant. This questionnaire included demographics, socioeconomic factors, details about the index case and degree of exposure, previous medical history, presence of symptoms, history of BCG immunization and presence of a scar, clinical findings, TST results, chest X-ray findings and the result of the QFT-GIT.

During the study period and until 2016, the BCG vaccination policy in the country included universal immunization of children at school entry regardless of the presence of risk factors. The BCG-Denmark vaccine has been used since the introduction of immunization. Since most of the children with a positive close contact history during the study period were born in Greece (283/314, 90.7%), there were very few immunized children ≤ 5 years (only 2/107). For this reason, the study population was restricted to children > 5 years of age (207 children in total) (Figure 1).

The standard dose of 2 TU PPD RT23 (Statens Serum Institut, Copenhagen, Denmark) was used for the TST. The QFT-GIT was conducted according to the manufacturer’s instructions at our research laboratory (Cellestis Limited, Carnegie, Victoria, Australia). The proposed research study was conducted in accordance with the amended Declaration of Helsinki and was approved by the hospital’s Ethics Review Board (02/02/2008, Protocol No. 1463).

Children who were born in another country or with at least one parent who was born in another country will be referred to as children in immigrant families (CIF) in the following text.

### 2.2. Setting

Our TB clinic is a reference center for pediatric TB for the greater Athens area, which has a population of about 3.8 million people according to the most recent estimate of the Hellenic Statistical Authority [17]. Pediatric TB cases are also referred from other central and southern parts of the country. Families are referred to our TB clinic for evaluation when an adult infectious TB case is diagnosed. Home visits for contact investigations are not conducted. In some cases, children are referred because of a positive TST after initial investigation at a local clinic. There are also instances where the source case is identified after a child is diagnosed with active or latent TB because of symptoms or during screening, respectively.

### 2.3. Statistical Analysis

Nominal variables are presented using absolute and relative frequencies. To evaluate the association between TB infection and patient characteristics, the chi-square test of independence and the Mann–Whitney test were used. To estimate the factors independently associated with TB infection, multiple logistic regression was applied. TB infection was the outcome variable. Dependent variables included demographic characteristics and those found to be statistically significant in the univariate analysis. Results are presented with odds ratios (OR) and 95% confidence intervals (95% CI).

*p*-values were considered significant if <0.05. All statistical analyses were performed with version 20 of the SPSS program (SPSS Inc., Chicago, IL, USA).

## 3. Results

### 3.1. Demographic, Socioeconomic and Clinical Characteristics

In total, 207 children >5 to 16 years of age who had known recent exposure to an adult with pulmonary TB and fulfilled the inclusion criteria were enrolled in the study. Median age was 10 years (interquartile range 7.4–12.4), and males represented about half of the sample (50.3%). Although most participants were born in Greece (88.4%), it was estimated that 55% of the enrolled subjects were Greek nationals, while 40.5% were CIF mostly originated from Albania, the Russian Federation and Eastern Europe. About half of the children enrolled (47.8%) had been immunized with the BCG vaccine, and there was documentation of BCG scar in 89 subjects (89.9%). Overall, 117/207 (56.5%) of participants tested positive with QFT-GIT.

Data regarding parent’s birthplace, living conditions, parent’s educational status and number of other children infected from the same source case are shown in Table 1.

### 3.2. Risk Factors for TB Infection

The association of participants’ characteristics with TB infection in the univariate analysis is shown in Table 2. Statistically significant associations were observed between TB infection and immigration (*p* = 0.001), mother’s educational level (*p* = 0.003), the number of other children infected by the index case (*p* = 0.001) and prior BCG vaccination (*p* = 0.002). A higher TB infection rate was recorded among CIF (70.2% vs. 47.2%). Among a number of socioeconomic factors, only maternal education was found to have a significant association with TB infection. Contacts born to mothers with higher (>12 years) education had a lower rate of LTBI (37.5% vs. 62.5%). Known transmission of the infection to other children in contact with the index case was a strong risk factor for TB infection. LTBI was diagnosed in 76.9% of exposed children when there was another infected child contact and in 72% of cases when more than one child was infected. Remarkably, LTBI was significantly more common among exposed unvaccinated children (68%) compared to those with prior BCG immunization (44.4%) (*p* = 0.002) (Table 2).

### 3.3. Multivariate Logistic Regression Analysis

Logistic regression was performed in order to assess the effect of multiple factors on the rate of TB infection. Factors that were significantly associated with TB infection in the univariate analysis were entered in the model. The results of this analysis are described in Table 3.

Crude logistic regression analysis revealed that BCG-vaccinated subjects had a 59% lower risk to present with TB infection after close contact with an adult index case compared with unvaccinated subjects (OR = 0.41, 95% CI: 0.23–0.73, *p* = 0.002). After adjustment for possible confounders, the protective effect of prior BCG immunization was estimated at 68%, with a range between 34% and 85% (OR = 0.32, 95% CI: 0.15–0.66, *p* = 0.002).

Other risk factors for TB infection confirmed in the multivariate analysis included history of immigration and transmission of infection to other exposed child contacts. Children in immigrant families exposed to an adult index case were more than twice as likely to be infected (OR = 2.27, 95% CI: 1.13–4.53, *p* = 0.021). The risk of infection was 4.6 times higher when there was another infected child contact and almost 5 times higher if more than one other child was infected.

## 4. Discussion

In this cross-sectional study, a large number of children older than 5 years of age closely exposed to an adult with infectious TB were examined with the aim of identifying risk factors for latent TB infection. A considerable number of close contacts took part in the study, and only those for whom there was thorough information about the contact history were included. It was shown that previous BCG immunization provides strong protection against TB infection to exposed children. Although the BCG vaccine was introduced about 100 years ago, it has only recently become evident from an increasing number of studies among contacts of active TB cases that BCG also provides protection against TB infection. A better understanding of the protective effect of BCG against infection is important for the use of this vaccine to improve TB control, as well as for the development of new vaccines against TB and for the design of new vaccine studies [13,18].

In a systematic review and meta-analysis of 14 studies in which TB infection was diagnosed with the use of IGRAs, BCG protective efficacy among children exposed during outbreaks or in the household was estimated at 19% (95% confidence interval 8% to 29%) [14]. Restriction of the analysis to a smaller number of studies that included information on progression to active tuberculosis at the time of screening showed a 27% protective efficacy against infection. Another study was conducted several years ago with the collaboration of five European centers (including our center) that are members of the Pediatric Tuberculosis Network European Trials (PTBNET) [13]. BCG protection against TB infection diagnosed with the use of QFT-GIT among 491 household contacts was estimated at 48% (95% confidence interval 19% to 67%). A large prospective study (INFECT study) was recently conducted among TB contacts in Indonesia, which is a high-prevalence country where children are immunized at birth [19]. It was shown that BCG vaccination provided strong protection against TB infection. Immunized children and adolescents 5 to 18 years of age had a significantly lower risk of a positive baseline IGRA (prevalence ratio 0.76; 95% CI: 0.67–0.87). It is likely that a proportion of these participants had already been infected during previous exposure. The risk of IGRA conversion at 14 weeks post-exposure in participants found negative at initial examination was reduced even further among those who were immunized (relative risk 0.37; 95% CI: 0.22–0.67). Protection declined with increasing age, and there was no protection among immunized contacts >38 years of age. It was also shown that protection decreased with increasing exposure. BCG protective efficacy was higher in the current study and was estimated at 59% in the univariate analysis and at 68% (95% CI: 34–85%) after controlling for potential confounders. This is higher than that previously reported and is comparable to that found in the Indonesian study described above. It is not clear whether these differences can be ascribed to methodological differences, age at BCG immunization or use of different vaccine strains. It is important to mention that until 2016 and during the study period, the BCG vaccination policy included school-aged children (5–6 years of age) regardless of risk factors. The short time interval between BCG vaccination and contact history may justify the high rate of BCG protection in older children in our study, since the median age of the subjects enrolled was 10 years. Future prospective studies of TB contacts in different settings may be able to define the degree of protection with higher accuracy.

In addition to the lack of BCG immunization, coming from an immigrant family and transmission of infection to other child contacts were predictors of TB infection. Transmission of infection to other children was the strongest risk factor indirectly indicating high infectivity of the index case. Previous studies have reported that younger age, origin from a TB endemic country, smear-positive disease and sputum smear grade, the presence of cough and cumulative exposure time are risk factors for transmission of TB infection to children after contact with an infectious case [19,20,21,22]. In highly TB endemic settings, identified predictive factors for LTBI include younger patient age, ethnicity, low maternal education and low socioeconomic status, whereas, in low TB endemic settings, the highest incidence rate of LTBI is found among recent immigrants [23,24]. TB incidence among recent immigrants usually reflects the epidemiology at the country of origin. Poor housing conditions and overcrowding in migrant shelters are documented risks for TB. Children in immigrant families are also at high risk for TB, and they may share similar vulnerabilities with foreign-born children. Pour living conditions, home visits by relatives or traveling to the country of origin with high TB incidence are all considered as risk factors for communicable diseases like TB [25].

## 5. Limitations

This study has a number of limitations. There may be a selection bias because children were not referred during contact investigations held officially by health departments during home visits. Children who sought care in our center were referred from different central and southern parts of the country; the results of sputum examination were often unknown or not available, or the index case was hospitalized in another country and the family had no information. Therefore, it was not possible to examine the infectivity of each source case. Despite the above limitations, it is unlikely that they had any effect on the comparison between BCG-immunized and non-immunized subjects. Finally, the source population was restricted to older children who were immunized at school entry and not at birth as in most similar studies. Therefore, the study findings cannot be extrapolated to younger ages or to children immunized at birth.

## 6. Conclusions

In conclusion, we were able to determine a strong protective role of BCG vaccination among children older than 5 years, immunized at school entry, who had close contact with an adult infectious TB case. Given the incomplete control of TB, especially in high-endemicity settings, the role of BCG immunization should be reappraised in light of these findings, and optimization of BCG use is warranted. Longitudinal contact studies are required in settings using different immunization schedules and vaccine strains in order to fully characterize vaccine effectiveness. There is a need for further studies to better define the degree of protection provided by BCG vaccination, especially to children under 5 years of age, who were not included in this report. It is also imperative to better understand the immunologic mechanisms behind this protection.

## Figures and Tables

**Table 1:** Demographic and clinical characteristics of subjects enrolled (N = 207).

Demographic/Clinical Parameters	Greek Nationals + Minorities * N (%)	CIF N (%)	Total N (%)	*p*-Value
**No. of subjects**	114 (55%) + 9(4.3%)	84 (40.5%)	207 (100%)	
**Median age (IQR)**	10.6 years (8–12.8)	9.8 years (6.5–12.1)	10 years (7.4–12.4)	0.076
**Females**	66 (53.7%)	37 (44.0%)	103 (49.7%)	0.174
**Birthplace (child/father)**				
Albania	-	7 (8.3%)/24 (28.6%)	7 (3.4%)/24 (11.6%)	
Russian Federation	-	2 (2.4%)/18 (21.4%)	2 (1%)/18 (8.7%)	
Eastern Europe	-	10 (11.9%)/18 (21.4%)	10 (4.8%)/18 (8.7%)	
Western Europe	-	2 (2.4%)/0	2 (1%)/0	
Asia	-	1 (1.2%)/8 (9.5%)	1 (0.5%)/8 (3.9%)	
Africa	-	2 (2.4%)/6 (7.1%)	2 (1%)/6 (2.9%)	
**Mother’s Education**				**0.001**
≤6 years	15 (12.2%)	14 (16.7%)	29 (14%)	
7–12 years	62 (50.4%)	60 (71.4%)	122 (59%)	
>12 years	46 (37.4%)	10 (11.9%)	56 (27%)	
**Residence**				0.383
House	63 (51.2%)	36 (42.9%)	99 (47.8%)	
Apartment	55 (44.7%)	42 (50.0%)	97 (46.8%)	
Other	5 (4.1%)	6 (7.1%)	11 (5.3%)	
**No. of rooms**				**0.001**
1	6 (4.9%)	11 (13.1%)	17 (8.2%)	
2	56 (45.5%)	56 (66.7%)	112 (54.1%)	
>2	61 (49.6%)	17 (20.2%)	78 (37.6%)	
**Frequency of contact**				0.114
Household	58 (47.2%)	49 (58.3%)	107 (51.7%)	
Non-household	65 (52.8%)	35 (41.7%)	100 (48.3%)	
**BCG vaccinated**	62 (50.4%)	37 (46.8%)	99 (49.0%)	0.620
**BCG scar**				0.227
Yes	56 (90.3%)	33 (89.2%)	89 (89.9%)	
No	3 (4.8%)	4 (10.8%)	7 (7.1%)	
Not recorded	3 (4.8%)	0	3 (3.0%)	
**TST ≥ 5 and <10**	39 (31.7%)	24 (28.6%)	63 (30.4%)	0.630
**TST ≥ 10**	84 (68.3%)	60 (71.4%)	144 (69.6%)	0.629
**QFT-GIT positive**	58 (47.2%)	59 (70.2%)	117 (56.5%)	0.001
**No. of other children infected**				0.295
0	66 (53.7%)	38 (45.2%)	104 (50.2%)	
1	41(33.3%)	37 (44.0%)	78 (37.6%)	
>1	16 (13.0%)	9 (10.7%)	25 (12%)	

* Roma or Muslim; IQR, interquartile range; CIF, children in immigrant families; BCG, Bacille Calmette–Guérin; TST, tuberculin skin test; QFT-GIT, QuantiFERON-TB Gold In-Tube test.

**Table 2:** Associations between participants’ characteristics and tuberculosis (TB) infection.

Participant’s Characteristics	TB Infection (Positive QFT-GIT Assay)		
	No N = 90 (%)	Yes N = 117 (%)	*p*-Value
**Age (years), median (IQR)**	106 (8.0–12.4)	10.0 (7.0–12.5)	0.539
**Sex**			0.951
Male	45 (43.3%)	59 (56.7%)	
Female	45 (43.7%)	58 (56.3%)	
**CIF**			**0.001**
No	65 (52.8%)	58 (47.2%)	
Yes	25 (29.8%)	59 (70.2%)	
**Μinority**			0.201
Yes	3 (33.3%)	6 (66.6%)	
**Residence**			0.282
House	40 (40.4%)	59 (59.6%)	
Apartment	47 (48.5%)	50 (51.5%)	
**No. of rooms**			0.265
1	10 (58.8%)	7 (41.2%)	
2	44 (39.3%)	68 (60.7%)	
>2	36 (46.2%)	42 (53.8%)	
**No. of inhabitants in the household, median (IQR)**	4 (3–5)	4 (4–4)	0.959
**Mother’s Education**			**0.003**
≤6 years	12 (41.4%)	17 (58.6%)	
7–12 years	43 (35.2%)	79 (64.8%)	
>12 years	35 (62.5%)	21 (37.5%)	
**No. of other children infected**			**0.001**
0	65 (62.5%)	39 (37.5%)	
1	18 (23.1%)	60 (76.9%)	
>1	7 (28%)	18 (72%)	
**BCG vaccination**			**0.002**
No	35 (34%)	68 (66%)	
Yes	55 (55.6%)	44 (44.4%)	

QFT-GIT, QuantiFERON-TB Gold In-Tube test; IQR, interquartile range; CIF, children in immigrant families; BCG, Bacille Calmette–Guérin.

**Table 3:** Multiple logistic regression of factors associated with TB infection among children > 5 years of age exposed to an infectious adult index case.

Demographic/Clinical Factors	% of TB Infected	OR (95% CI)	*p*-Value
**Crude regression**			
**BCG vaccination**			
No	66.0%	Ref. cat.	
Yes	44.4%	0.41 (0.23–0.73)	**0.002**
**Adjusted regression**			
**Age**		1.07 (0.95–1.19)	0.264
**Female gender vs. male**	56.3% vs. 56.7%	1.05 (0.55–2.01)	0.870
**BCG vaccination**			
No	66.0%	Ref. cat.	
Yes	44.4%	0.32 (0.15–0.66)	**0.002**
**CIF vs. non-CIF**	70.2% vs. 47.2%	2.27 (1.13–4.53)	**0.021**
**Mother’s Education**			
≤6 years	58.6%	Ref. cat.	
7–12 years	64.8%	1.44 (0.55–3.75)	0.313
>12 years	37.5%	0.65 (0.23–1.89)	0.658
**No. of children infected**			
0	37.5%	Ref. cat.	
1	76.9%	4.62 (2.27–9.39)	**0.001**
>1	72%	4.72 (1.70–13.09)	**0.003**

TB, Tuberculosis; BCG, Bacille Calmette–Guérin; CI, confidence interval; OR, odds ratio; Ref. cat., reference category; CIF, children in immigrant families.
